# Perivascular epithelioid cell tumor of the rectum: report of a case and review of the literature

**DOI:** 10.1186/1477-7819-12-12

**Published:** 2014-01-13

**Authors:** Amane Kanazawa, Shoichi Fujii, Ten-i Godai, Atsushi Ishibe, Takashi Oshima, Tadao Fukushima, Mitsuyoshi Ota, Norio Yukawa, Yasushi Rino, Toshio Imada, Junko Ito, Akinori Nozawa, Munetaka Masuda, Chikara Kunisaki

**Affiliations:** 1 Gastroenterological Center, Yokohama City University Medical Center, 4-57 Urafune-cho Minami-ku, Yokohama-shi, Kanagawa-ken 232-0024, Japan; 2 Department of Surgery, Yokohama City University, 3-9 Fukuura, Kanazawa-ku, Yokohama-shi, Kanagawa-ken 236-0004, Japan; 3 Department of Pathology, Yokohama City University Medical Center, 4-57 Urafune-cho Minami-ku, Yokohama-shi, Kanagawa-ken 232-0024, Japan

**Keywords:** Perivascular epithelioid cell tumor, PEComa, Transanal endoscopic microsurgery

## Abstract

We report a case of perivascular epithelioid cell tumor arising in the rectum of a 55-year-old woman. The tumor was treated by transanal endoscopic microsurgery. After 1 year follow-up, the patient is alive with no radiologic or endoscopic evidence of recurrence. Perivascular epithelioid cell tumor is a rare mesenchymal tumor characterized by co-expression of melanocytic and smooth muscle markers. This rare tumor can arise in various organs, including the falciform ligament, uterus, uterine cervix, liver, kidney, lung, breast, cardiac septum, pancreas, prostate, thigh, and gastrointestinal tract. Perivascular epithelioid cell tumor of the gastrointestinal tract is very rare, with only 23 previously reported cases. We review the literature on perivascular epithelioid cell tumors arising in the gastrointestinal tract.

## Background

Perivascular epithelioid cell tumors (PEComas) were defined as “mesenchymal tumours composed of histologically and immunohistochemically distinctive perivascular epithelioid cells” by the World Health Organization [1]. PEComas classically include a wide spectrum of entities, such as angiomyolipoma (AML), lymphangioleiomyomatosis (LAM), and clear-cell “sugar” tumors (CCST) of the lung. PEComas other than AML, LAM, and CCST are very rare mesenchymal tumors that have been referred to as PEComa-not otherwise specified (PEComa-NOS) [1,2]. A review of 51 cases of PEComa-NOS revealed that 41% of reported cases of PEComa-NOS originated in the uterine corpus [3].

Gastrointestinal PEComa-NOS are very rare. To our knowledge, 23 cases have been reported in the literature [4-22]; consequently, the clinical and biological characteristics of PEComa-NOS are poorly understood. We report a case of PEComa arising in the rectum and review the clinicopathological characteristics of gastrointestinal PEComa-NOS.

## Case presentation

A 55-year-old woman was referred to our hospital because of a rectal submucosal tumor detected on a colonoscopic examination. She had undergone a sigmoidectomy for sigmoid colon carcinoma 2 years previously in our hospital. Postoperatively, the final disease stage was T1N0M0 (Stage I) according to the TNM classification. The histological subtype of the sigmoid colon cancer was moderately differentiated adenocarcinoma. There had been no evidence of recurrence or metastasis during 2 years follow-up. The patient showed no signs of tuberous sclerosis complex.

**Figure 1:**
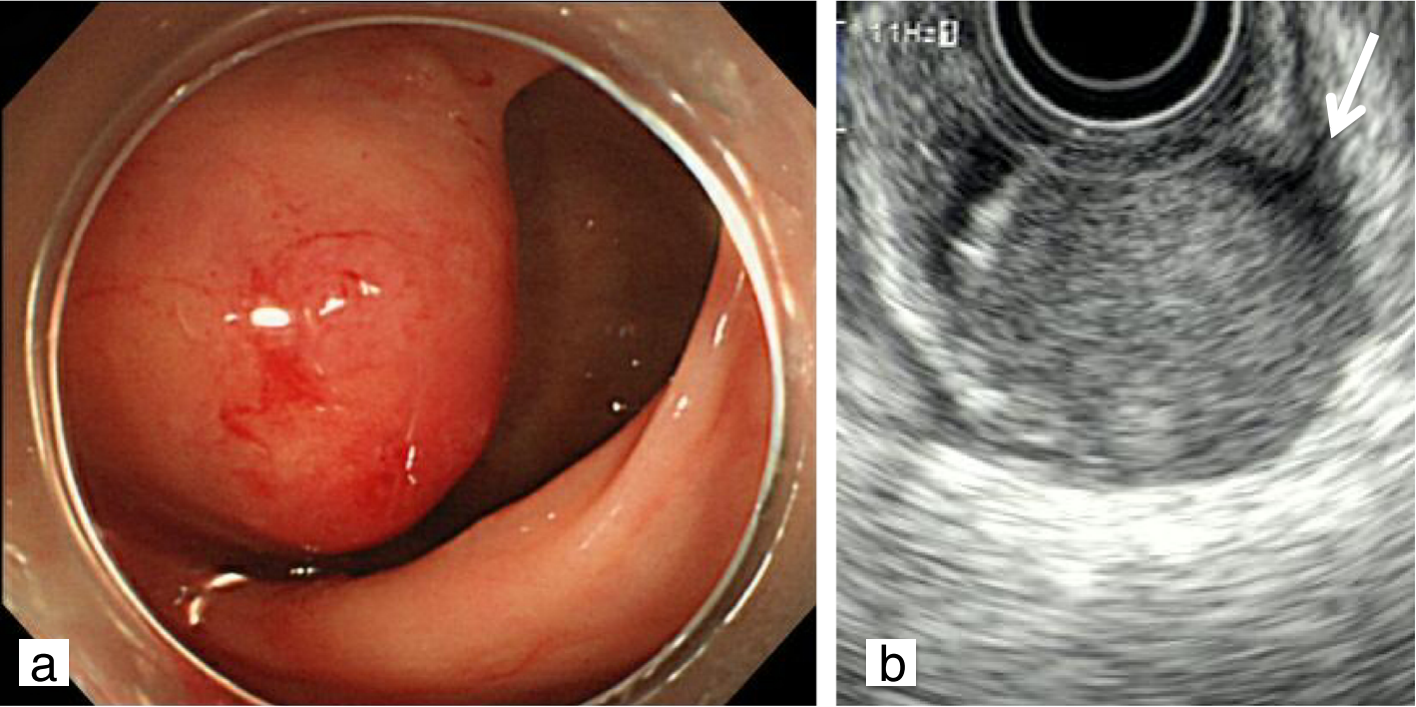
**Findings on colonoscopy and endoanal ultrasound scanning. (a)** Colonoscopic examination showed a submucosal tumor 3 cm in diameter arising in the posterior rectal wall. **(b)** Endoanal ultrasound scanning showed a heterogeneous, low-echoic tumor with a nodular appearance arising in the muscularis (long arrow) of the posterior rectal wall.

On digital rectal examination, a hard mass was palpated on the posterior wall of the rectum. The results of other physical examinations of the abdomen and other organs were normal. Laboratory data showed no marked abnormalities, including tumor markers such as carcinoembryonic antigen and carbohydrate antigen 19-9. Colonoscopic examination in our hospital revealed a submucosal tumor 3 cm in diameter arising in the posterior rectal wall, 10 cm from the anal verge (Figure 1a). The submucosal tumor was accompanied by an ulcer and superficial mucosal bleeding at its center. A biopsy was performed, but a histopathological diagnosis could not be established. An endoanal ultrasound scan showed a heterogenous, low echoic, nodular tumor arising in the muscularis of the posterior rectal wall (Figure 1b). The tumor was suspected to invade the muscularis propria and subserosa. Contrast-enhanced computed tomography of the chest, abdomen, and pelvis detected no lymph node lesions around the rectum or metastasis to other organs.

The patient underwent transanal endoscopic microsurgery for a suspected diagnosis of rectal stromal tumor. We excised the full-thickness of the rectal wall excision with a 1 cm safety margin of normal mucosa around the tumor. The wound in the rectal wall was closed with a running suture. Macroscopically, the resected specimen showed a brown mass 2.5 cm in maximum diameter, with a central ulcer, an ill-defined border, and no capsule formation. Surface ulceration was prominent in association with absence of the rectal mucosa, which was ascribed to a prior endoscopic biopsy.

**Figure 2:**
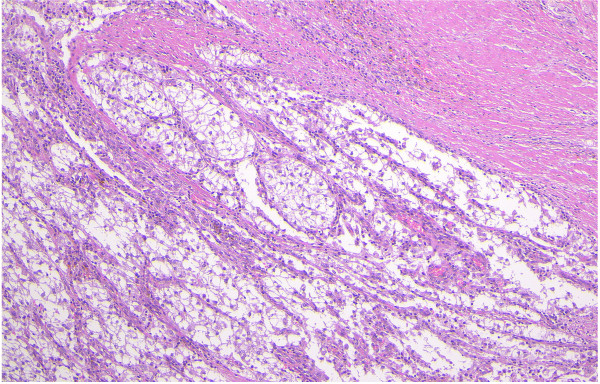
**Appearance of tumor stained with hematoxylin and eosin.** The tumor consisted of round and polygonal cells with clear-to-eosinophilic granular cytoplasm. The tumor cells proliferated in a honeycomb-like fashion.

On microscopic examination, the tumor resided mainly in the musclaris propria and invaded the mucosa and was exposed on its surface. Histopathologically, the tumor consisted of round and polygonal cells with clear-to-eosinophilic granular cytoplasm. The tumor cells proliferated in a honeycomb-like appearance (Figure 2). The tumor cell nuclei showed hyperchromasia, nuclear enlargement, and prominent nucleoli. The tumor cells were negative for Fontana-Masson staining. Periodic acid-Schiff stain-positive intracytoplasmic granules, which were digested by diastage, were noted. The tumor cell showed slight to moderate nuclear atypia, with no mitosis or tumor necrosis. There was no lymphatic or vascular invasion.

**Figure 3:**
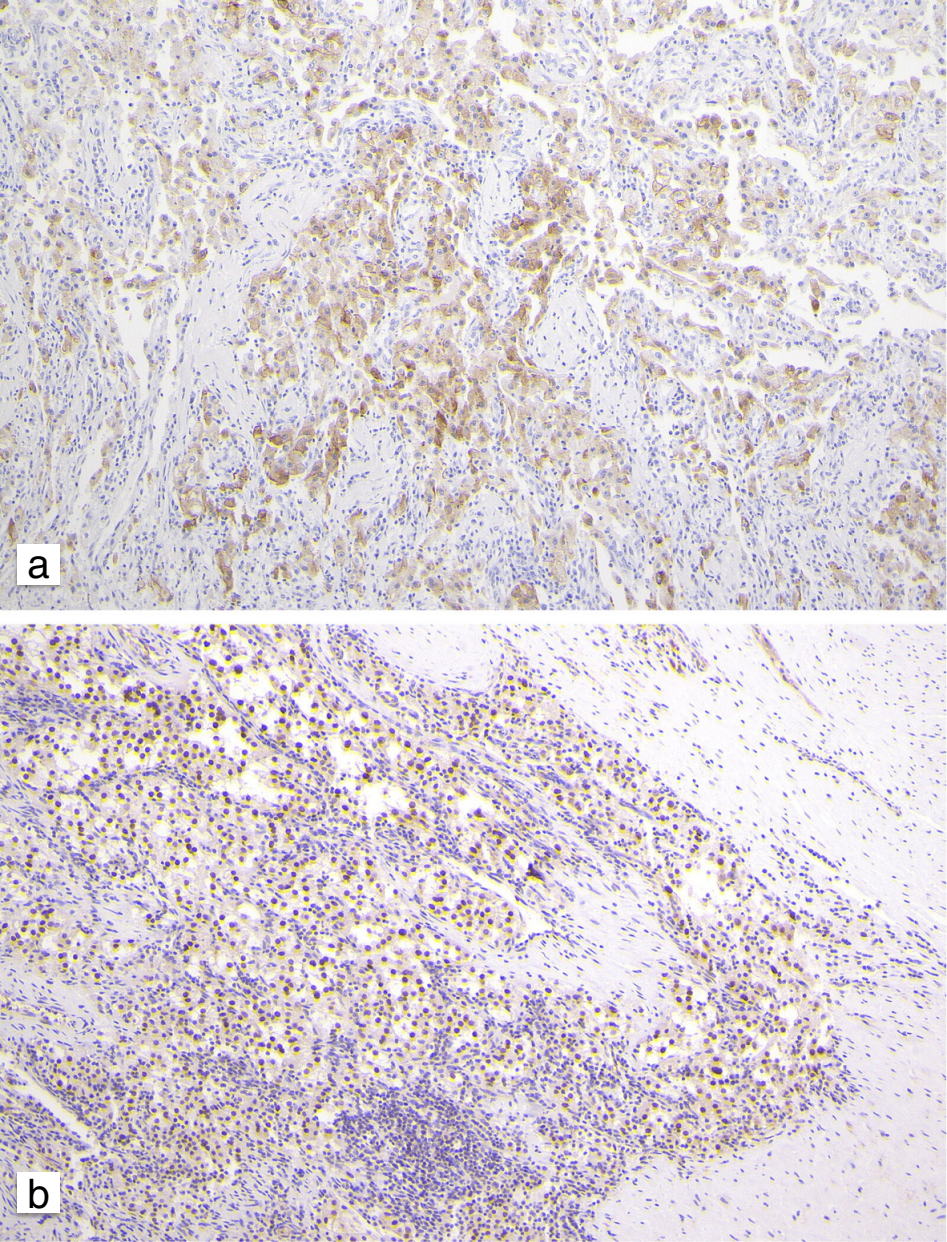
**Immunohistochemical findings of the tumor. (a)** HMB-45 is positive in the cytoplasm of the tumor cells. **(b)** Nuclear expression of TFE3 is observed in the tumor cells. HMB-45, melanoma-associated antigen; TFE3, transcription factor E3.

Immunohistochemically, the tumor cells were positive for melanoma-associated antigen (HMB-45), neuron-specific enolase, CD68, and transcription factor E3 (TFE-3), but negative for AE1/AE3, CAM5.2, epithelial membrane antigen, smooth muscle actin, Desmin, MyoD1, Caldesmon, Calponin, Synaptophysin, Chromogranin A, NCAM, Vimetin, CD34, S-100, Melan-A, CD99, CD10, D2-40, CD138, P-ALP, and multiple myeloma oncogene-1 (Figure 3ab). The proliferation marker Ki-67 showed a nuclear positivity in approximately 15% of cells. An intestinal PEComa was diagnosis on the basis of these findings. This patient received only surgical resection. She is undergoing regular surveillance and remains free of disease at 15 months after operation.

## Discussion

PEComas are part of a group of very rare mesenchymal neoplasms. Bonetti and colleagues [23] were the first to propose the concept of the perivascular epithelioid cell in 1992. The term PEComa was coined by Zamboni and colleagues [24] in 1996 to describe this rare family of lesions. In 2002, the World Health Organization accepted the designation PEComa as a distinct mesenchymal neoplasm consisting of histologically and immunohistochemically unique perivascular epithelioid cells [1]. This rare tumor has been reported in various organs, such as the falciform ligament, uterus, uterine cervix, liver, kidney, lung, breast, cardiac septum, pancreas, prostate, thigh, and gastrointestinal tract. PEComas usually show immunoreactivity for both melanocytic (HMB-45 and/or melan-A) and smooth muscle (actin and/or desmin) markers. Recently, several reports described TFE3 expression in PEComas [14,22,25]. In one review, TFE3 was positive for 3 of 24 cases of gastrointestinal PEComa. In our case, positivity for TFE3 contributed to the diagnosis of PEComa.

In this study, we focused on gastrointestinal PEComa-NOS and did not include classic AML and LAM of the gastrointestinal tract in our review. The clinicopathological features of the PEComa-NOS included in this study are summarized in Table 1. The ratio of males to women was 8 to 16, suggesting that primary gastrointestinal PEComa-NOS is more common in females, similar to PEComa-NOS arising in other organs. Mean age at the diagnosis of primary gastrointestinal PEComa-NOS was 31.5 years (range 7 to 63 years). The mean diameter of primary gastrointestinal PEComa-NOS was 45.5 mm (range 12 to 100 mm). The tumor was located in the colon in 14 patients (58.3%), rectum in 5 (20.8%), the small bowel in 2 (8.3%), the duodenum in 2 (8.3%), and the stomach in 1 (4.3%). Surgical resection was performed in nearly all patients, and only two received adjuvant chemotherapy. One patient was given adjuvant interferon-α2b therapy, and the other case received adjuvant chemotherapy with doxorubicin, ifosfamide, and mesna in accordance with the Children’s Oncology Group non-rhabdomyosarcoma soft tissue sarcoma protocol. Neither of these patients had any evidence of recurrence. Follow-up data were available for 19 patients, and the median follow-up was 15 months (range 3 to 41 months) after surgery. Three cases had recurrence [5,12], two of whom underwent re-resection of their tumors. The 3-year disease free survival rates of the 19 patients was 75%. Two patients died of their disease. One patient died 3 months after surgery, and the other died 38 months after the first operation.

**Table 1:** Patient characteristics and tumor morphologic and histopathological features and outcomes

**Year**	**Author [reference]**	**Age (years)**	**Sex**	**Location**	**Size (mm)**	**Infiltrative border**	**Mitosis (/50 HPF)**	**LVI**	**Tumor necrosis**	**Treatment**	**AC**	**Follow-up**
2001	Tazelaar *et al.*, [4]	9	F	Rectum	30	-	Rare	-	-	Trans anal resection	-	NER at 14 months
2001	Tazelaar *et al.*, [4]	40	F	Rectum	80	-	Rare	-	-	Excision	-	NER at 6 months
2003	Yanai *et al*., [5]	32	F	Jejunum	75	-	n.a	-	+	Partial resection of jejunum	-	Pelvic wall recurrence at 13 months, Right ovary recurrence at 25 months
2004	Birkahaeuser *et al.*, [6]	35	F	Cecum	35	n.a	Few	n.a	-	Right hemicolectomy	-	n.a
2004	Genevay *et al.*, [7]	38	F	Cecum	35	n.a	Rare	n.a	-	Right hemicolectomy	n.a	n.a
2005	Evert *et al.*, [8]	58	F	Rectum	80	n.a	286	n.a	+	n.a	n.a	n.a
2005	Yamamoto *et al.*, [9]	43	F	Descending	80	n.a	2	+	+	Partial colectomy	-	Peritoneal dissemination at 20 months, DOD at 38 months
2005	Mhanna *et al.*, [10]	15	M	Duodenum	45	+	Low	n.a	-	Pancreaticoduodenectomy	-	NER at 24 months
2006	Baek *et al.*, [11]	16	F	Transverse	25	n.a	0	n.a	-	Endoscopic resection	-	NER at 24 months
2006	Agimy *et al.*, [12]	63	F	Ileum	45	n.a	13	n.a	+	Partial resection of ileum	-	Abdominopelvic recurrence at 14 months
2008	Narayanaswamy *et al.*, [13]	34	M	Duodenum	35	+	n.a	-	-	Duodenectomy	-	NER at 18 months
2008	Cho *et al.*, [14]	16	F	Transverse	18	n.a	n.a	-	-	n.a	-	NER at 41 months
2008	Pisharody *et al.*, [15]	11	M	Sigmoid	12	n.a	Rare	n.a	n.a	Partial colectomy	-	NER at 5 months
2008	Righi *et al.*, [22]	11	M	Sigmoid	35	+	Rare	n.a	+	Segmental resection	-	n.a
2009	Ryan *et al.*, [16]	15	F	Rectum	37	-	2	-	+	Low anterior resection	+	NER at 9 months
2009	Qu *et al.*, [17]	43	F	Cecum	20	-	3	-	-	Right hemicolectomy	-	NER at 25 months
2010	Park *et al.*, [18]	7	M	Ascending	37	n.a	n.a	n.a	n.a	Right hemicolectomy	+	NER at 26 months
2010	Shi *et al.*, [19]	38	F	Sigmoid	60	n.a	n.a	n.a	-	Segmental resection	-	NER at 8 months
2010	Shi *et al.*, [19]	42	M	Ascending	45	n.a	n.a	n.a	-	Segmental resection	-	NER at 15 months
2010	Shi *et al.*, [19]	38	F	Descending	48	n.a	n.a	n.a	-	Segmental resection	-	NER at 32 months
2010	Shi *et al.*, [19]	45	M	Ascending	35	n.a	n.a	n.a	-	Segmental resection	-	NER at 36 months
2010	Freeman *et al.*, [21]	17	F	Sigmoid	60	-	Low	n.a	n.a	Partial resection	-	NER at 15 months
2012	Waters *et al.*, [20]	42	M	Gastric	100	n.a	n.a	n.a	n.a	Distal gastrectomy	-	DOD at 3 months
2012	Present case	55	F	Rectum	25	+	0	-	-	Trans anal resection	-	NER at 12 months

*/50 HPF* 50 high-power fields, *AC* adjuvant chemotherapy, *DOD* died of disease, *F* female, *LVI* lymphovascular invasion, *M* male, *n.a* no data available, *NER* no evidence of recurrence.

Although the treatment of choice for gastrointestinal PEComa is surgical resection, the overall management strategy for gastrointestinal PEComa remains to be established. Furthermore, potential benefits of adjuvant chemotherapy have not been investigated. In a recent study of PEComa of soft tissue and gynecologic origin, Folpe and colleagues [25] suggested criteria for classifying PEComas into benign, uncertain malignant potential, and malignant categories. They proposed that malignancy was predicted by the presence of two of the following findings: tumor size greater than 5 cm, infiltrative tumor border, high nuclear grade and cellularity, more than 1 mitosis/50 high-power fields, tumor necrosis, and vascular invasion. In our patient, the tumor was classified as low grade according to these diagnostic criteria because there was an infiltrative tumor border, but none of the other findings.

Given the present situation, close follow-up, including imaging studies and colonoscopy, is mandatory after surgical resection of gastrointestinal PEComa, especially in patients with high-grade malignancy. Because the outcomes of gastrointestinal PEComa remain unclear, further long-term studies in larger number of patients are needed.

## Conclusions

We reported a rare case of rectal PEComa and reviewed the clinicopathological characteristics of gastrointestinal PEComa-NOS. At the present time, the most effective treatment for gastrointestinal PEComa is surgical resection. But the postoperative management including adjuvant chemotherapy still has not been established. So patients with postoperative PEComa-NOS should be carefully followed up.

## Consent

Written informed consent was obtained from the patient for publication of this case report and accompanying images. A copy of the written consent is available for review by the Editor-in-Chief of this journal.

## Abbreviations

AML: Angiomyolipoma; CCST: Clear-cell “sugar” tumors; HMB-45: Melanoma-associated antigen; LAM: Lymphangioleiomyomatosis; PEComa: Perivascular epithelioid cell tumor; PEComa-NOS: PEComa-not otherwise specified; TFE3: Transcription factor E3.

## Competing interests

The authors declare that they have no competing interests.

## Authors’ contributions

AK, SF, TG, AI, TO, and TF performed the surgical treatment. AK and SF drafted the manuscript. TI and AN performed the pathological studies. NY, YR, TI and MM revised this manuscript. CK critically reviewed the manuscript and gave final approval for publication. All authors have read and approval the final manuscript.
